# Construction and evaluation of an efficient C‐Jun siRNA to downregulate matrix metalloproteinase in human keratinocytes and fibroblasts under UV exposure

**DOI:** 10.1002/mgg3.1047

**Published:** 2019-11-14

**Authors:** Hong Xiao, Ruinian Yang, Fang Yang, Yanan Zhao, Yin Liu

**Affiliations:** ^1^ Department of Plastic Surgery The Second Affiliated Hospital of Kunming Medical University Kunming Yunnan China; ^2^ Department of Plastic Surgery YESTAR Aesthetic Plastic Hospital Kunming Yunnan China

**Keywords:** C‐Jun, collagen, matrix metalloproteinase, photoaging, siRNA, ultraviolet radiation

## Abstract

**Background:**

C‐Jun and EGFR have not been explored as targets via the mechanism of RNA silencing**.** Hence, this study designed an efficient C‐Jun‐h‐825 small interfering RNA (siRNA) and investigated its effect on matrix metalloproteinase (MMP) and collagen expression in human keratinocytes exposed to UV radiation.

**Methods:**

Five C‐Jun siRNAs were designed and screened for their ability to downregulate C‐Jun expression in human fibroblasts. These constructs were used to study changes in skin cancer‐related protein expression. HaCaT cells were grouped into 5‐carboxyfluorescien (FAM‐labeled) C‐Jun‐h‐825 siRNA + 2 hr prior irradiation; mock transfected + 2 hr prior irradiation; normal control; irradiation only for 2 hr; and Blank. Twenty‐four hours posttransfection, mRNA and protein levels of MMP‐I, MMP‐III, collagen‐I and collagen‐III were determined using standard RT‐PCR and ELISA kits.

**Results:**

FAM‐labeled C‐Jun siRNA showed 80%–90% transfection efficiency. There was a significant increase in MMP‐I and MMP‐III and decrease in Col‐I and III mRNA levels when the cells were exposed to UV irradiation without siRNA transfection compared to blank (*p* < .05). This effect was reversed upon transfection with C‐Jun‐h‐825 (*p* < .01).

**Conclusion:**

Thus, C‐Jun‐h‐825 siRNA might help restore skin collagen by decreasing MMP expression in cells exposed to UVA. Constructs and vectors designed herein have the potential to be translated into a treatment for photoaging induced skin cancer.

## Graphical abstract

C‐Jun siRNA has been designed in this study. C‐Jun siRNA helped restore skin collagen by decreasing MMP expression in cells exposed to UVA. Constructs and vectors designed herein have the potential to be translated into a treatment for photoaging induced skin cancer.

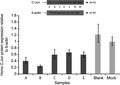

## 1 INTRODUCTION

Currently, skin cancer is the most common malignancy, and organ transplant patients and people who use tanning beds show an elevated risk of developing the condition. It contributes to a significant burden in terms of morbidity and economic costs for the affected population (Gordon, 2013). Although the incidence of skin cancer is much higher in the Caucasian population, recent reports show that its prevalence is steadily increasing in the Asian population as well. Basal Cell Carcinoma, caused by extensive UV exposure, is the most common type of skin cancer detected in the Chinese population (Kim, Del Rosso, & Bellew, 2009).

A variety of complex intracellular processes contribute to the initiation of malignant skin lesions, and the epidermal growth factor receptor (EGFR)‐mediated signal transduction pathway has been implicated in this process (Rodust, Stockfleth, Ulrich, Leverkus, & Eberle, 2009). EGFR signaling results in the activation of cellular kinases such as mitogen‐activated protein kinases (MAPKs) and extracellular signal–regulated Kinases (ERK), which are known to play a crucial role in skin cancer initiation and progression. One of the consequences of EGFR signal transduction is the phosphorylation of the activator protein‐1(AP‐1) transcription factor subunit, C‐Jun (Uribe & Gonzalez, 2011). AP‐1 activation and binding is known to play a role in upregulating several genes, including the MMPs (matrix metalloproteinases), which are involved in collagen breakdown under UV exposure (Pittayapruek, Meephansan, Prapapan, Komine, & Ohtsuki, 2016). By degrading the collagen extracellular matrix (ECM) of skin exposed to ultraviolet (UV) radiation, MMPs facilitate neoplastic cell migration and metastasis. Inhibition of these cellular processes is likely to limit neoplastic transformation and carcinogenesis, and several strategies that target these pathways are being deployed for the treatment of skin cancer. Among these, a range of anti‐EGFR agents and Tyrosine Kinase Inhibitors (TKIs) such as Gefitinib and Erlotinib are extensively used, along with anti‐EGFR antibodies such as Cetuximab (Khan, Alam, & Yoo, 2011; Spallone, Botti, & Costanzo, 2011).

Recent advances in genetic technology have led to the exploitation of cellular RNA‐silencing pathways in order to provide a permanent and transmissible solution to the problem of neoplasty (Wang, Rao, Senzer, & Nemunaitis, 2011). These include the use of siRNA (silencing RNA) and small hairpin RNA (shRNA)‐based interventions, which rely on existing RNA‐based silencing pathways in order to prevent the expression of target proteins by designing small, specific double‐stranded RNA molecules complimentary to the mRNA of the protein. These molecules are then processed into their single‐stranded form by intracellular machinery comprising of the RISC (RNA‐induced silencing complex) and subsequently prevent the translation of target mRNAs by annealing to them through base complementarity (Morris, 2008; Wei et al., 2011). Currently, several siRNA and shRNA molecules are being developed for the treatment of skin cancer. However, *C‐JUN* (HGNC:6204) and *EGFR* (HGNC:3236) have not been explored as targets via the mechanism of RNA silencing (Bogusławska & Małecki, 2013).

In this work, we aim to design a highly efficient C‐Jun siRNA molecule and study its ability to downregulate MMP expression under UV radiation exposure in vitro in human keratinocytes and fibroblasts. In addition, to improve the probability of gene knockdown in vivo, we design a plasmid vector for the transfection of human *C‐Jun* and *EGFR* shRNA.

## 2 MATERIALS AND METHODS

### 2.1 Ethical compliance

The study was approved by the ethics committee of the second affiliated hospital of Kunming medical University.

### 2.2 siRNA design

The complete sequence of the *homo sapiens C‐Jun* gene was obtained from the publicly available Entrez gene database (GenBank) provided by NCBI. Shared transcript sequences to which siRNAs could be designed were selected from the complete sequence. Starting from the AUG initiator codon in the target gene, sequences were searched and 19 base sequences at its 3′ end were found to be potential siRNA target sites and were noted down (bases at the 3′ UTR end were also selected, as the fraction of functional siRNAs in this region was similar to that of the ORF). The selected target sequence was parsed to generate a list of candidate siRNAs. The effective siRNA sequence with low stability at base position 9–14 at the 5′ end of antisense strand was considered as the interference target. Based on this, sequences were chosen to cover the greatest possible range of properties and values such as GC content between 30% and 52%: 15th and 19th positions in the sense strand contain at least three A/U bases; excluding the reverse and repeated sequences, the 3rd and 19th bases in the sense strand would be A; the 10th base in the sense strand would be U; the 19th base in the sense strand would not be G/ C; and the 13th base in the sense strand would not be G.

Candidate sequences overlapping with unusable positions in the target, breaching the above criteria were discarded. Each candidate sequence was scored upon satisfying the aforesaid properties on the basis of compliance of general rules of RNA interference and the target sequence loci were selected. One point was deducted for those failing to satisfy the aforesaid items and score one for every A/ U appearing from position 15th–19th base of sense strand. The RNA sequences with a score greater than 6 were considered effective.

### 2.3 Fibroblast culture and transfection

Human skin fibroblasts were taken from patient's sample and a written informed consent was taken for the same. Furthermore, the cells were grown in six‐well culture plates using DMEM supplemented with 10% inactivated fetal bovine serum (FBS), 100 μg/ml ampicillin trihydrate, and 100 μg/ml streptomycin. On day 2, the medium was replaced with D‐Hank's solution. At 80%–90% confluency, the nutrient solution was aspirated from the culture plates and washed twice with 2 ml of D‐Hank's solution. Cells were trypsinized with 2‐ml trypsin EDTA solution and were incubated at 37°C for 3–5 min. Then, 2 ml of DMEM nutrient solution containing 10% FBS was added and the cells were allowed to form a single cell suspension. Cells were counted using a blood counting chamber and were diluted to 1.5 × 10^6^ cells/ml, when required. The cells were inoculated in the six‐well plates at a concentration of 1.5 × 10^6^ cells/well and incubated at 37°C with 5% CO_2_ for 24 hr. One hundred and fifty microliter of DEPC‐H_2_O was used to dissolve each siRNA (with OD 260 nm) and a final concentration of 20 μM was used in further experiments. Exactly 250 μl of Opti‐MEM I was added to a 1.5 ml eppendorf tube followed by the addition of 5 μl of siRNA. Furthermore, 5 μl of lipofectamine 2,000 at room temperature was added for 20–30 min. Medium in the –six‐well plates were drained and 1 ml of DMEM nutrient solution was added to each well. Transfection medium was added drop wise into the six‐well plates and incubated for 4–6 hr after which the transfection medium was drained and 1.5 ml of DMEM containing 10% of FBS was added and the cells were allowed to divide at 37°C with 5% CO_2_.

### 2.4 SDS‐PAGE and western blotting

Lysates were obtained by directly adding 80 μl of cell lysis buffer to the cells. After heating and sonication, proteins were separated by SDS‐PAGE and transferred onto a nitrocellulose membrane using a semi‐dry blot system. After the transfer, 1× Ponceau S solution was used to stain the membrane. This was followed by blocking, which was performed overnight at 4°C in TBS. The membrane was then washed thrice with 1× TBST for 10 min each. Incubation with both primary (C‐Jun polyclonal antibody—E254) and secondary antibodies were performed at room temperature for 1 hr in the same solution as the blocking. After each antibody incubation, blots were washed in TBS. SuperSignal West Pico Chemiluminescent Substrates were used for the chemical lighting detection and exposed to the X‐ray films. Images were taken using the Gel‐Pro analyzer software.

### 2.5 HaCaT cell culture

Immortalized human HaCaT keratinocytes were procured from Kunming Animal Research Center of China Scientific College after approval from institutional ethical committee. The cells were then maintained as a monolayer in Dulbecco's modified Eagle's medium (DMEM), supplemented with 10% FBS, 50 units/ml ampicillin, and 50 μg/ml streptomycin. The cells were incubated at 37°C, in a humidified chamber with 5% CO_2_.

### 2.6 siRNA transfection and UV irradiation

Cells were inoculated in a 10 cm^2^ dish at a density of 3–4  ×  10^5^/ml and cultured for 24 hr until 80% confluency was reached. Cells were separated into five groups based on transfection and irradiation status as: (a) C‐Jun‐h‐825 siRNA (4 µg); (b) mock transfected (10 µl lipofectamine 2,000); (c) normal control (only transfection, no irradiation); (d) irradiation only (15 J/cm^2^); (e) Blank (not irradiated and not transfected). Except for the cells in groups C and E, all cells were irradiated with 15 J/cm^2^ UV radiation using the UV irradiator (NS‐8F; Sanwa Medical) emitting in the UVA spectral region (320–400 nm) with maximum emission at 365 nm. After 2 hr of irradiation, cells in groups C were transfected with siRNA without exposure to UV. Cells were covered with serum and DMEM medium during irradiation and were transferred to fresh DMEM after irradiation. The C‐Jun siRNA was coupled with fluorescent label, 5‐carboxyfluorescein (FAM) at the sense strand to track the transfection in HaCaT cells by fluorescent microscopy. Group D cells were not transfected and group A cells were only transfected with lipofectamine 2,000 without prior UVA irradiation. Six hours after transfection, the culture media were replaced and the cells were allowed to incubate for 24 hr at 37°C in 5% CO_2_. The cell viability of fibroblast by UV irradiation was assessed using MTT assay.

### 2.7 RNA extraction and real‐time PCR analysis

RNA was extracted by treating the transfected cells with Trizol. About 1 ml of the reagent was added to the cells and centrifuged for 5 min at room temperature followed by the addition of 0.2‐ml chloroform and vigorous shaking for 15 s. The centrifuge tubes were allowed to stand at 4°C for 2–3 min and centrifuged at 1,200*g* for 15 min. The supernatant was collected in a separate tube and was washed with 70% ethanol. Another two washes were carried out at a centrifugation of 8,000*g* for 15 s and the silica membrane of the centrifugation column was dried after each wash. Next, the supernatant was transferred to a 1.5‐ml RNase free centrifuge tube. Around 40 μl of water was added and the contents were allowed to stand at room temperature for 1–5 min, then centrifuged at 8,000*g* for 1 min after which RNA was eluted. Total RNA extracted from each sample (5 μl) was reverse transcribed using 0.2 μl (2 U/μl) Moloney Murine Leukemia Virus reverse transcriptase. The reaction was started with the addition of a reverse transcriptase (RT) primer (1 μM), 5‐μl RNA template, and 4 μl of 5× RT buffer at 42°C for 45 min or 85°C for 10 min. Real‐time PCR analysis was carried out using the SYBR^®^ Green ExScriptTM PCR Kit. Twenty micromolar each of forward and reverse primer with a final volume of 0.1 μl were added along with rTaq DNA polymerase (5 U/ml) (Table S1).

### 2.8 Homo C‐Jun

Forward primer 5′‐AAGTGAAAACCTTGAAAGCTCAG‐3′.

Reverse Primer 3′‐TTAACGTGGTTCATGACTTTCTG‐5′.

### 2.9 Homo 5s RNA

Forward primer 5′‐ACGGCCATACCACCCTGAAC‐3′.

Reverse Primer 3′‐GGCGGTCTCCCATCCAAGTA‐5′.

The reaction was carried out at 95°C for 3 min; 95°C for 12 s and 62°C for 40 s for a total of 40 cycles. The housekeeping gene 5s RNA was used as amplification control. A ΔCT method was then used to process the data and to calculate the relative gene expression.

### 2.10 Cell lysates and ELISA

Lysates were obtained by the direct addition of 80 μl of cell lysis buffer onto the cells and the levels of MMP‐I, MMP‐III, collagen‐I, and Collagen‐III proteins were determined using commercially available kits. Absorbance was measured at 450 nm by a SpectraMax^®^ 190 microplate reader and the result was analyzed by Soft max Pro5.2 software.

### 2.11 Statistical analysis

Statistical analyses were performed on SPSS 13.0 software. The ΔCT calculations were performed for quantifying mRNA expression levels from the RT‐PCR analysis. The transfection efficiency, mRNA and protein expression levels were compared between groups using the one‐way analysis of variance (ANOVA).

## 3 RESULTS

### 3.1 C‐Jun siRNAs transfected into human fibroblasts significantly downregulated C‐Jun protein expression

Based on the properties of an ideal siRNA sequence, we designed and screened five siRNAs which satisfied all criteria. The sequence of these five siRNAs (21 base pairs each) is given in Table S2. Data obtained from RT‐PCR analysis of C‐Jun mRNA levels 48 hr following siRNA transfection for 5 siRNA constructs (at a dose of 20 μM) are summarized in Table 1. Change in concentration was calculated using the ΔCT method and revealed that the silencing of C‐Jun was highest in JUN‐h‐825 siRNA transfected fibroblasts with 76% efficiency relative to 5s rRNA expression. JUN‐hm‐1866 had the lowest efficiency among the 5 siRNAs screened. Mock transfected and scrambled siRNA samples were used as negative controls and confirmed the specificity of the silencing effect.

**Table 1:** C‐Jun knockdown efficiency in human dermal fibroblasts for different siRNAs

siRNA	Mean CT, *n* = 3 (C‐Jun)	Mean CT, *n* = 3 (5s RNA)	ΔCT	ΔΔCT	2−ΔΔCT	% KD
A	26.84 ± 0.32	11.20 ± 0.05	15.64 ± 0.33	1.32 ± 0.36	0.40 (0.31–0.51)	60
B	28.07 ± 0.05	11.69 ± 0.11	16.38 ± 0.12	2.06 ± 0.19	0.24 (0.21–0.27)	76%
C	26.29 ± 0.33	11.21 ± 0.06	15.08 ± 0.34	0.76 ± 0.37	0.59 (0.46–0.76)	41%
D	26.53 ± 0.06	11.62 ± 0.08	14.91 ± 0.10	0.59 ± 0.17	0.66 (0.59–0.75)	34%
E	26.38 ± 0.13	11.30 ± 0.08	15.09 ± 0.15	0.77 ± 0.20	0.59 (0.51–0.68)	41%
Blank	25.28 ± 0.29	11.24 ± 0.06	14.04 ± 0.30	−0.28 ± 0.33	1.22 (0.97–1.53)	−78%
Mock transfected	25.47 ± 0.12	11.15 ± 0.06	14.32 ± 0.14	0.00 ± 0.20	1.00 (0.87–1.14)	0%
Nontargeting siRNA	25.57 ± 0.22	10.95 ± 0.11	14.62 ± 0.24	0.30 ± 0.28	0.81 (0.67–0.99)	19%

Abbreviations: ΔCT, difference between both mean CTs; ΔΔCT, difference between both ΔCTs; 2−ΔΔCT, fold change in expression; KD, knockdown.

**Figure 1:**
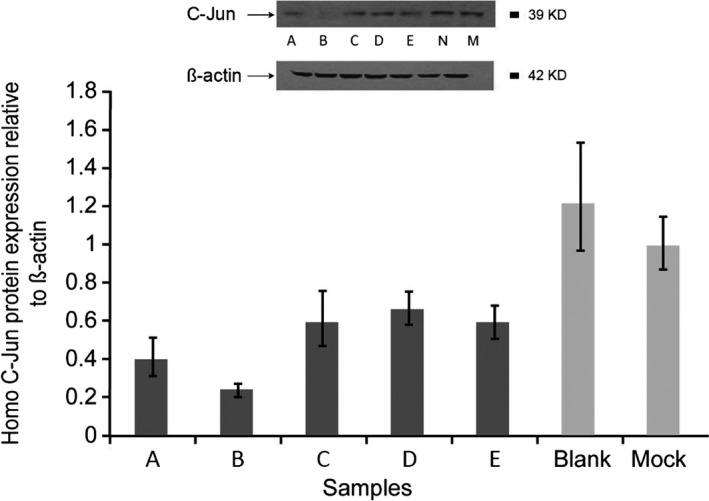
C‐Jun protein expression in human skin fibroblasts transfected with siRNA. Screening of siRNA for gene knockdown efficiency in fibroblasts. siRNA from clones 1–5 was used in order to transfect fibroblasts. Protein expression (figure) and mRNA levels (graph) both showed that siRNA from clone 2 was most effective at reducing target gene expression. β‐Actin was used as the loading control

Protein Expression was studied using Western blotting. Expression of C‐Jun was found to be the lowest in the JUN‐h‐825 siRNA sample and highest in the JUN‐hm‐1866 siRNA sample, indicating that the JUN‐h‐825 siRNA was the most efficient. Normal levels of C‐Jun protein expression were observed in the mock and untreated samples. β‐Actin was used as a loading control (Figure 1).

### 3.2 UV irradiation of the fibroblasts and keratinocytes

The results of MTT assay after irradiation at 15 J/cm^2^ revealed satisfactory preservation of cell viability although morphological changes were noted. The irradiation dose in the current study was selected based on the internal standardization performed with the fibroblasts and HaCaT cell lines (data not shown).

### 3.3 siRNA transfection into HaCaT cells was highly efficient

**Figure 2:**
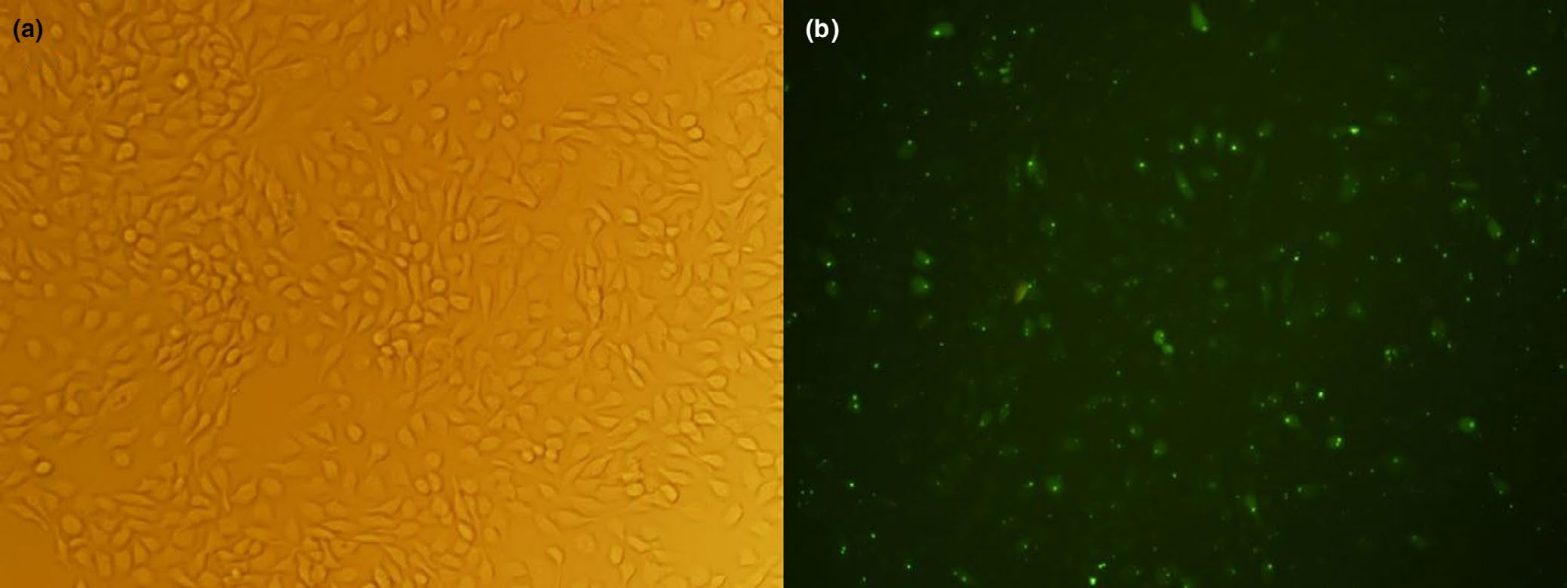
FAM‐labeled C‐Jun siRNA transfected HaCaT cells. Transfection efficiency in HaCaT cells. (a) showing siRNA transfected HaCaT cells in rear light microscope at 40× magnification; (b) showing FAM‐labeled C‐Jun siRNA transfected HaCaT cells in fluorescence microscope at 40× magnification

Counting of HaCaT cells transfected with FAM‐labeled C‐Jun siRNA was carried out using fluorescence microscopy. An 80%–90% transfection efficiency was found. Bright field and fluorescent images of FAM positive C‐Jun siRNA cells at 40× magnification are shown in Figure 2a and b.

### 3.4 Human fibroblast MMP‐I/III and Collagen‐I/III protein levels

**Figure 3:**
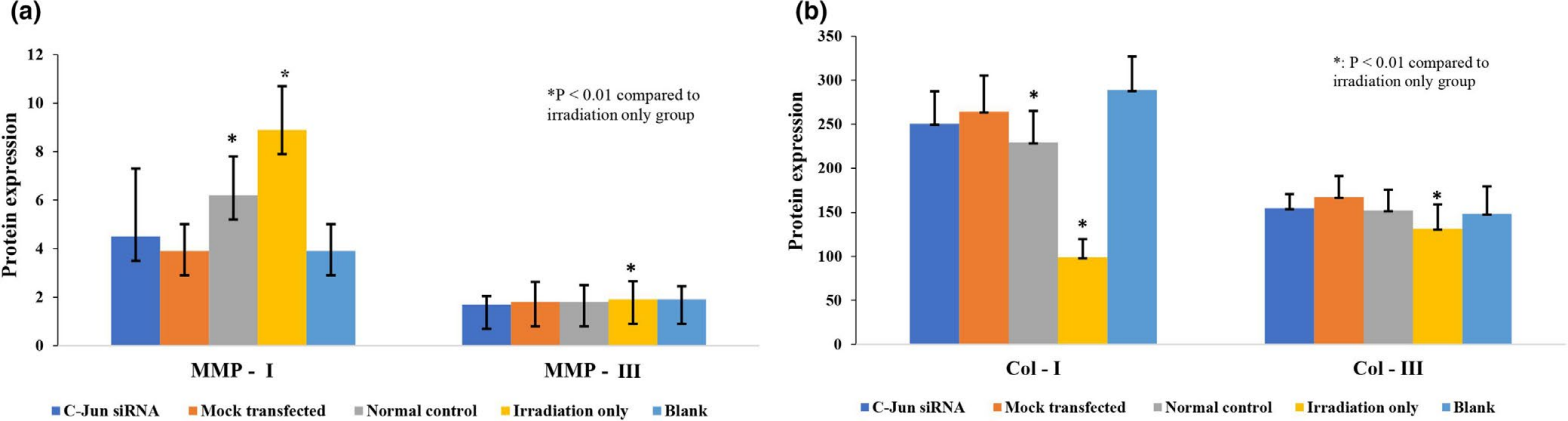
Protein expression using ELISA in human fibroblast cells. (a) Protein expression in MMP‐I and MMP‐III; (b) Protein expression in Collagen‐I and Collagen‐III. *p < .01 compared to irradiation only group

Protein levels of MMP‐I and MMP‐III were significantly higher in UV‐irradiated cells without transfection (*p* < .01), whereas protein levels of Col‐I and Col‐III were significantly lower in them (Figure 3).

### 3.5 HaCaT cell MMP‐1 (HGNC:7155)/3 (HGNC:7173) and Collagen‐1 (HGNC group ID 439)/III (HGNC:2201) gene expression patterns and protein levels change significantly upon C‐Jun siRNA transfection and exposure to UV radiation

Changes in mRNA levels of MMP‐I, MMP‐III, Collagen‐I and Collagen‐III were measured using quantitative RT‐PCR (ΔCT) in UV‐irradiated HaCaT cells transfected with C‐Jun siRNA or scrambled siRNA (mock transfection). ΔCT values showed that there was a significant increase in the MMP‐I and MMP‐III mRNA levels when the cells were exposed to UV irradiation without siRNA transfection compared to cells which were not UV irradiated (*p* < .05). MMP levels significantly dropped in cells irradiated and transfected with C‐Jun siRNA (levels were similar to cells which were not UV irradiated) compared to the un‐transfected, UV‐irradiated group (*p* < .01). Also, the drop in MMP levels was significantly greater in the UV‐irradiated siRNA transfected cells compared to normal control and mock‐transfected cells (*p* < .05, Table S3).

ΔCT values also showed that there was a significant decrease in collagen‐I and collagen‐III mRNA levels when un‐transfected cells were exposed to UV irradiation compared to cells which were not UV irradiated (*p* < .05). Conversely, these levels increased significantly in UV‐irradiated siRNA transfected cells (to levels similar to cells which were not UV irradiated) compared to un‐transfected cells which were UV irradiated (*p* < .01). Also, the rise in collagen levels was significantly greater in the UV‐irradiated siRNA transfected cells compared to normal control and mock‐transfected cells (*p* < .05, Table S4).

**Figure 4:**
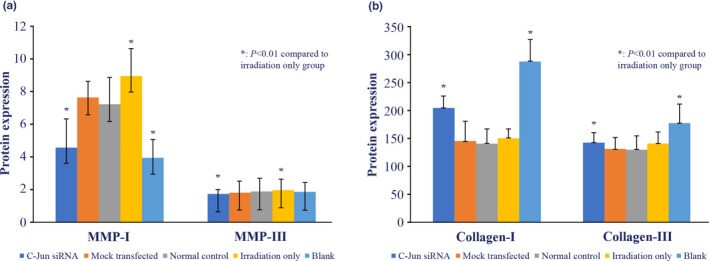
Protein expression using ELISA in HaCaT cell line. (a) Protein expression in MMP‐I and MMP‐III; (b) Protein expression in Collagen‐I and Collagen‐III. *p < .01 compared to irradiation only group

Protein expression was validated using an ELISA. Results showed that MMP‐I and MMP‐III protein levels were significantly lower in UV‐irradiated cells transfected with C‐Jun siRNA compared to UV‐irradiated un‐transfected cells (*p* < .01). Similarly, the expression of type I and III collagen in these cells was higher compared to un‐transfected, UV‐irradiated cells (*p* < .01) (Figure 4). These results are consistent with mRNA expression data.

The cell viability of fibroblast by MTT assay showed that the optical density (OD) value decreased with an increase in UVA dose. Compared with the control group (0.61 ± 0.32), the OD value of UVA 10 J/cm^2^ (0.51 ± 0.34) and UVA 20 J/cm^2^ (0.22 ± 0.03) decreased significantly (*p* < .01).

## 4 DISCUSSION

In this work, the effect of C‐Jun downregulation using RNA interference technology on skin cancer‐related protein expression was studied in human fibroblasts and keratinocytes. We successfully designed a highly efficient C‐Jun siRNA construct and through a series of in vitro experiments demonstrated that its downregulation inhibits MMP expression while simultaneously increasing collagen expression in human fibroblasts and keratinocytes exposed to UV radiation. Previously, Curran and Franza showed that C‐Jun and C‐Fos dimerize via their leucine zipper regions to form the nuclear transcription factor AP‐1, which binds to the promoter region of target genes such as MMPs and thereby regulates their expression (Curran & Franza, 1988).

Several downstream pathways regulated by C‐Jun participate in the process of skin aging. Reactive oxygen species (ROS)‐mediated damage (generated upon UV exposure) induces a negative regulator of collagen production, cysteine‐rich protein 61 (CCN1) which promotes the loss of type I collagen. Functional blocking of C‐Jun has been shown to significantly reduce CCN1 expression and preserve the collagen content of the skin (Qin et al., 2014). Several anti‐oxidants have been developed in order to combat skin aging by preserving collagen content (Boisnic, Branchet‐Gumila, & Nocera, 2005; Mohapatra et al., 2016). In addition, several naturally occurring flavonoids have also been studied for their ability to treat photoaging mediated by the C‐Jun AP‐1/MAPK pathway (Li, Liu, Chen, Sun, & Wang, 2007; Lim & Kim, 2007; Shim, Kwon, Han, & Hwang, 2008). Another proposed mechanism is that UV exposure activates *EGFR* which in turn activates the extracellular‐regulated kinases (ERK1/2), subsequently activating *C‐Jun* which promotes the degradation of skin collagen (Chauhan & Shakya, 2009). As the *C‐Jun* part of the AP‐1 transcription factor was found to be a common intermediate involved in all signaling cascades related to skin aging, C‐Jun transcription factor specific targets are likely to be able to hinder several contributing downstream events and may prove beneficial for sustained skin protection. siRNAs, which are RNA duplexes of 21–23 nucleotides in length provide new insights into the functions of the targeted gene in biological systems and facilitate mRNA destruction in a RISC (RNA‐induced silencing complex)‐dependent manner (Elbashir, Lendeckel, & Tuschl, 2001). Currently, no siRNA‐based therapies which target the process of UV‐induced photoaging have been designed.

In this study, we designed six siRNAs targeting the C‐Jun transcription factor based on practical guidelines for the construction of an ideal siRNA and screened for the siRNA with the highest silencing efficiency. Upon transfection into dermal fibroblasts, the expression of C‐Jun was knocked down by >40% in all siRNAs except the JUN‐hm‐1866 siRNA. The JUN‐h‐825 siRNA achieved 76% knockdown in the RT‐PCR analysis. Hence, the most efficient siRNA, Jun‐h‐825 was used for further analysis of MMP and collagen expression in our work. There was a significant drop in the MMP‐I and MMP‐III levels along with a proportionate increase in the collagen content in the cells treated with JUN‐h‐825 siRNA compared to the irradiated group without siRNA transfection. In previous studies, UV dose ranging from 5J/m^2^ to 20J/m^2^ was used in multiple cell types for estimating the effect on EGFR signaling. Since there is no single recommended UV dosage for multiple cell types, we performed standardization experiments in laboratory (data not shown) and evaluated different UV dosages on the cell viability, transfection efficiency, and siRNA stability within UV‐irradiated cells. Further MTT assay was performed for multiple UV dosages and we established a cell viability of more than 90% to be associated with evaluable transfection efficiency. Thus, UV exposure at 15 J/m^2^ for 2 hr was selected for subsequent experiments. These findings were similar to a recent study that reported a suppression of *C‐Jun* expression by siRNAs and regulation of MMP expression to inhibit aberrant endothelial and neo‐vascular growth (Zhang, Fahmy, diGirolamo, & Khachigian, 2006). Moreover, the gross effects of the C‐Jun siRNA may depend on the differential expression of collagen in fibroblasts and keratinocytes. But since, the potential therapeutic effect is reflected by the combined effects of C‐Jun siRNA in both keratinocytes and fibroblast, we did not account for the differential expression in our study. But, since fibroblasts play a predominant role in collagen synthesis, we performed siRNA standardization in fibroblasts cells to identify the optimum siRNA in our study. While we compared collagen and MMP expression in fibroblasts and keratinocytes transfected with and without C‐Jun siRNA, we did not compare the differential effects in keratinocytes and fibroblasts (UV irradiated, C‐Jun siRNA‐transfected fibroblasts vs. UV irradiated, C‐Jun siRNA‐transfected keratinocytes). Furthermore, these findings are consistent with those published in the 29th annual meeting of NESPS, where in vitro and in vivo preclinical study on the use of topical C‐Jun siRNAs to prevent photoaging in Americans was reported. The NESPS abstract concluded that cutaneous siRNA delivery prevented UV‐induced expression of *C‐Jun* and MMP and the associated changes to collagen, skin elasticity, and tensile strength, ultimately retaining the features of nonirradiated skin, and the novel, gene‐specific strategy may be rapidly translatable to clinical use for photoaging (Hwang et al., 2012). In light of the findings from the present study and from the NESPS abstract, we demonstrate a potentially translatable efficiency of C‐JUN‐h‐825 siRNA for treating photoaging. This information warrants further in vivo studies and clinical trials in future in order to translate these findings for the effective treatment of skin cancer.

### 4.1 Strength and limitations

The study, however, has its limitations and the readers are expected to draw conclusions with caution. First, these findings are in vitro based and no in vivo animal models were subjected to the proposed therapy and hence, these results may vary in an animal's internal environment. Second, efficient delivery of siRNAs into the host requires a careful selection of carriers which is beyond the scope of our current work. However, as C‐Jun plays a role in skin aging via five crucial pathways viz., EGFR/ERK1/2, PDGFR/ERK1/2, MAPK/AP‐1, TNF‐alpha, and IL‐1 pathways, all of which lead to collagen degradation, targeting C‐Jun would be an efficient strategy to restore skin collagen and prevent aging (Kappelmann, Bosserhoff, & Kuphal, 2014). Furthermore, the effectiveness of the siRNA after dose‐ and time‐dependent UV irradiation may not be possible in transfection models and may need alternative experimental models for confirmation. This study was performed as a proof of concept experiment and further studies with more relevant skin cell lines for protein expression are recommended. However, there are no drugs targeting C‐Jun thus far. Given the advancements made in the use of siRNA technology, moving forward the C‐Jun siRNA validated in this study might translate into an effective therapy for the treatment of UV‐induced photoaging and skin cancer.

## 5 CONCLUSION

In conclusion, C‐Jun‐h‐825 siRNA might help restore skin collagen by decreasing MMP expression in skin cells exposed to UVA, which might be beneficial for the treatment of photoaging. In vivo studies and clinical trials are required to translate the benefits of JUN‐h‐825 proposed in this report into clinically feasible therapies for skin photoaging.

## CONFLICT OF INTEREST STATEMENT

The authors declare that they have no conflict of interest.

## AUTHORS’ CONTRIBUTIONS

HX conceived and design the study, coordination of the group, and writing the first draft. RY and FY participated in the search and resume of articles and evaluation of its quality and helped to draft the manuscript. YZ and YL participated in the design of the study, in evaluating the molecular aspects involve in the revision and in reviewing the draft. All authors read and approved the final manuscript.

## ETHICAL APPROVAL AND CONSENT TO PARTICIPATE

A written informed consent was obtained from patients.

## Data Availability

All the data associated with this study are given in the manuscript or the supplementary file.
