# Supplementary material for: Construction and evaluation of an efficient C‐Jun siRNA to downregulate matrix metalloproteinase in human keratinocytes and fibroblasts under UV exposure
Source: Mol Genet Genomic Med. 2019 Nov 14;8(1):e1047. doi: 10.1002/mgg3.1047 (PMC6978249; doi:10.1002/mgg3.1047)
Supplement: Supplementary file 1 [file MGG3-8-e1047-s001.docx]

**Supplementary Table 1. Forward and reverse primers for RT-PCR**

| **Gene Description** | **Primer** | **Sequences** |
| --- | --- | --- |
| Homo C - Jun | F primer | AAGTGAAAACCTTGAAAGCTCAG |
|  | R primer | TTAACGTGGTTCATGACTTTCTG  Size 103 base pairs |
| Homo 5s RNA | F primer | ACGGCCATACCACCCTGAAC |
|  | R primer | GGCGGTCTCCCATCCAAGTA  Size 91 base pairs |
