# Supplementary material for: Construction and evaluation of an efficient C‐Jun siRNA to downregulate matrix metalloproteinase in human keratinocytes and fibroblasts under UV exposure
Source: Mol Genet Genomic Med. 2019 Nov 14;8(1):e1047. doi: 10.1002/mgg3.1047 (PMC6978249; doi:10.1002/mgg3.1047)
Supplement: Supplementary file 2 [file MGG3-8-e1047-s002.docx]

**Supplementary Table 2. Five siRNAs which met the functionality criteria**

| **Gene** | **SiRNA sequence** |
| --- | --- |
| JUN-h-3287 (A) | GCAUUCUUGUCACAAUAAATT |
| JUN-h-825 (B) | CGGGAGGCAUCUUAAUUAATT |
| JUN-h-2584 (C) | AGCUGAUUACUGUCAAUAATT |
| JUN-hm-1866 (D) | GGAACAGGUGGCACAGCUUTT |
| JUN-hm-1930 (E) | CUAACGCAGCAGUUGCAAATT |
| Negative/antisense sequence | TTCTCCGAACGTGTCACGT |
