# Supplementary material for: Construction and evaluation of an efficient C‐Jun siRNA to downregulate matrix metalloproteinase in human keratinocytes and fibroblasts under UV exposure
Source: Mol Genet Genomic Med. 2019 Nov 14;8(1):e1047. doi: 10.1002/mgg3.1047 (PMC6978249; doi:10.1002/mgg3.1047)
Supplement: Supplementary file 3 [file MGG3-8-e1047-s003.docx]

**Supplementary Table 3. Expression of MMP-I and MMP-III mRNA in HaCaT cells**

| **Groups** | **Mean CTE, n=3** | **Mean CTC, n=3** | **ΔCT** | **ΔΔCT** | **2 - ΔΔCT** |
| --- | --- | --- | --- | --- | --- |
| **MMP-I mRNA expression** | | | | | |
| C-Jun siRNA | 23.87 ± 0.09 | 17.12 ± 0.10 | 6.75 ± 0.14 | 0.25 ± 0.19 | 0.84 (0.74 - 0.96)*^#^ |
| Mock transfected | 23.62 ± 0.10 | 17.12 ± 0.10 | 6.50 ± 0.14 | 0.00 ± 0.20 | 1.00 (0.87 - 1.15) |
| Normal control | 23.66 ± 0.08 | 17.12 ± 0.10 | 6.54 ± 0.13 | 0.04 ± 0.19 | 0.97 (0.85 - 1.11) |
| Irradiation only | 23.33 ± 0.12 | 17.12 ± 0.10 | 6.21 ± 0.16 | -0.29 ± 0.21 | 1.23 (1.06 - 1.42)^$^ |
| Blank | 23.97 ± 0.08 | 17.12 ± 0.10 | 6.85 ± 0.13 | 0.35 ± 0.19 | 0.79 (0.69 - 0.90) |
| **MMP-III mRNA expression** | | | | | |
| C-Jun siRNA | 17.23 ± 0.08 | 17.13 ± 0.16 | 0.10 ± 0.18 | 0.20 ± 0.26 | 0.87 (0.73 - 1.04)* |
| Mock transfected | 17.04 ± 0.07 | 17.13 ± 0.16 | -0.10 ± 0.18 | 0.00 ± 0.25 | 1.00 (0.84 - 1.19) |
| Normal control | 17.00 ± 0.14 | 17.13 ± 0.16 | -0.13 ± 0.22 | -0.03 ± 0.28 | 1.02 (0.84 - 1.24) |
| Irradiation only | 16.86 ± 0.05 | 17.13 ± 0.16 | -0.27 ± 0.17 | -0.17 ± 0.25 | 1.13 (0.95 - 1.34) |
| Blank | 17.40 ± 0.08 | 17.13 ± 0.16 | 0.27 ± 0.18 | 0.37 ± 0.26 | 0.77 (0.65 - 0.92) |

CTE: CT value of experimental group; CTC: CT value of GAPDH.

*P<0.01 compared to irradiation only group; #P<0.05 compared to mock transfected group and normal control; $P<0.05 compared to mock, normal control and blank
