# Supplementary material for: Construction and evaluation of an efficient C‐Jun siRNA to downregulate matrix metalloproteinase in human keratinocytes and fibroblasts under UV exposure
Source: Mol Genet Genomic Med. 2019 Nov 14;8(1):e1047. doi: 10.1002/mgg3.1047 (PMC6978249; doi:10.1002/mgg3.1047)
Supplement: Supplementary file 4 [file MGG3-8-e1047-s004.docx]

**Supplementary Table 4. Expression of Collagen-I and III mRNA in HaCaT cells**

| **Groups** | **Mean CTE, n=3** | **Mean CTC, n=3** | **ΔCT** | **ΔΔCT** | **2 - ΔΔCT** |
| --- | --- | --- | --- | --- | --- |
| **Collagen-I mRNA expression** | | | | | |
| C-Jun siRNA | 25.87 ± 0.20 | 16.84 ± 0.13 | 9.02 ± 0.24 | -0.29 ± 0.30 | 1.22 (0.99 - 1.50)* |
| Mock transfected | 26.16 ± 0.11 | 16.84 ± 0.13 | 9.31 ± 0.17 | 0.00 ± 0.25 | 1.00 (0.84 - 1.18) |
| Normal control | 26.25 ± 0.12 | 16.84 ± 0.13 | 9.41 ± 0.18 | 0.10 ± 0.25 | 0.93 (0.78 - 1.11) |
| Irradiation only | 26.13 ± 0.10 | 16.84 ± 0.13 | 9.29 ± 0.17 | -0.02 ± 0.24 | 1.02 (0.86 - 1.20) |
| Blank | 25.73 ± 0.07 | 16.84 ± 0.13 | 8.89 ± 0.14 | -0.42 ± 0.23 | 1.34 (1.14 - 1.57) |
| **Collagen-III mRNA expression** | | | | | |
| C-Jun siRNA | 23.84 ± 0.08 | 17.35 ± 0.06 | 6.49 ± 0.10 | -0.17 ± 0.12 | 1.13 (1.04 - 1.22)* |
| Mock transfected | 24.01 ± 0.01 | 17.35 ± 0.06 | 6.66 ± 0.06 | 0.00 ± 0.09 | 1.00 (0.94 - 1.06) |
| Normal control | 24.02 ± 0.11 | 17.35 ± 0.06 | 6.68 ± 0.12 | 0.02 ± 0.14 | 0.99 (0.90 - 1.09) |
| Irradiation only | 23.85 ± 0.04 | 17.35 ± 0.06 | 6.51 ± 0.07 | -0.15 ± 0.09 | 1.11 (1.04 - 1.19) |
| Blank | 23.77 ± 0.04 | 17.35 ± 0.06 | 6.42 ± 0.08 | -0.24 ± 0.10 | 1.18 (1.10 - 1.26) |

CTE: CT value of experimental group; CTC: CT value of GAPDH.

*P<0.01 compared to irradiation only group
